# Cashew Apples in Ghana: Stakeholders' Knowledge, Perception, and Utilization

**DOI:** 10.1155/2022/2749234

**Published:** 2022-01-06

**Authors:** Yaw Gyau Akyereko, Faustina Dufie Wireko-Manu, Francis Alemawor, Mary Adzanyo

**Affiliations:** ^1^Department of Food and Post-Harvest Technology, Faculty of Applied Science and Technology, Koforidua Technical University, Koforidua, Ghana; ^2^Department of Food Science and Technology, Faculty of Biosciences, KNUST, Kumasi, Ghana; ^3^GIZ, Accra, Ghana

## Abstract

Cashew (*Anacardium occidentale*), a crop projecting Ghana internationally beside cocoa, is usually cultivated for its nut, for both local and international markets. The cashew apple is underutilized in many African countries. This study is aimed at determining the knowledge, perception, and utilization of cashew apples in Ghana among stakeholders in the cashew value chain. Results from the study showed that the cashew industry consisted of farmers (89.1%), nut buyers (6.8%), extension officers (3.5%), and processors (0.6%) with uneven distribution of males (66.2%) and females (33.8%). Cashew apple utilization was low (<10%), though 84.37% had in-depth knowledge on the health benefits and value-added products made from the apples. Cashew apple is mainly utilized as fresh fruits or juice, with minor uses as an ingredient in food preparation, animal feed formulation, and production of mushroom, weedicide, ethanol, and manure. The cashew apple processors identified high cost of processing equipment, perishability of apples, lack of capital, market, technical know-how, and government support as challenges. Based on these findings, education of the stakeholders on value addition or processing of the apples at household and industrial levels and provision of stimulus packages to private processors are recommended to maximize cashew apple utilization in Ghana.

## 1. Introduction

Cashew (*Anacardium occidentale*) is a cash crop of the family *Anacardiaceae* and genus *Anacardium*, which grows well in Vietnam, India, Brazil, and most West African countries including Cote d'Ivoire, Tanzania, Guinea-Bissau, Ghana, Nigeria, and Benin. Cote d'Ivoire is the leading producer of cashews globally, followed by India and Vietnam [1]. Cashew was initially introduced into West Africa as an ornamental and afforestation tree, until when the nuts of the plant received international recognition for oil extraction and other value-added products. Ghana is performing well on the international market by contributing about 171,924 MT of nuts to world production volume in 2019 [2].

A lot of farmers in the middle belt of Ghana where cocoa is not doing well are into cashew production and obtain their livelihood from cashew and its activities like buying and selling of the nuts [3]. The cashew production industry in Ghana has received tremendous development and growth through interventional strategies like the supply of free seedlings, financial support and grants, research, education, and training workshops for farmers and other stakeholders by the government of Ghana, Nongovernmental Organizations (NGOs), and some benevolent institutions. Notwithstanding these successes, the cashew industry in Ghana is still faced with challenges like insect infestation, rainfall failure, price fluctuations, limited market, and utilization of cashew apples [4, 5].

Cashew apple, which consists of about 90% of the total weight of cashew fruit, has both medicinal and nutritional components such as vitamins, minerals, and other plant phytochemicals needed for growth and development, treatment of sore throat and chronic dysentery, and management of cardiovascular diseases and certain cancers [6]. The apple has found application in food systems as pure juice, juice blends, jam, syrup, pastries, ethanol, wine, and other value-added products in some advanced countries [7].

Sadly, studies have reported that only about 10% of the cashew apples are utilized as fruits for domestic consumption in Africa, and a greater percentage goes to waste on farmers' fields causing environmental nuisance due to limited knowledge, lack of processing and harvesting equipment, and high perishability, among others [7, 8]. Kolliesuah et al. [9] also revealed that cashew apple utilization by farmers and dwellers of cashew-growing communities in West African countries like Nigeria, Mali, Guinea-Bissau, and Benin is less than 10% due to the lack of technical know-how, high priority for export, limited processing, high astringency, and perishability of the apples, though production in these areas is very high. The study also acknowledged that Ghana is among the countries at the forefront of cashew production, but there is little utilization of the apples by its populace.

It is against this background that this study sought to assess the knowledge, perception, and utilization of cashew apples among the key stakeholders in the cashew value chain in Ghana, in order to identify major challenges or issues contributing to low utilization of the cashew apples in Ghana and proffer solutions for policymakers, researchers, and educational or training institutions to strengthen the cashew industry, income generation, and job creation.

## 2. Materials and Methods

### 2.1. Study Area and Sampling

The Bono, Bono East, and Ahafo regions of Ghana were purposively selected for this study. The three regions are well noted for the production and trading of cashew nuts in Ghana. Bono region is located on the longitude W 2° 16.450195 and latitude N 7° 53.481948 and shared boundaries with Savannah region, Ahafo, Bono East, and Cote d'Ivoire on the north, south, east, and west, respectively. Bono East also lies on longitude W 1° 49.5667 and latitude N 7° 51.1667 and shares borders with Savannah (north), Bono (west), Ashanti (south), and Volta Lake (east), and Ahafo region (W 2° 48.59631 and N 6° 51.472138) on the other side shares boundaries on the north and south with Bono region, Ashanti (east), and Western North (south) of Ghana. Nine districts, three from each region, Bono (Wenchi and Jaman North and South), Bono East (Kintampo South and Nkoranza North and South), and Ahafo (Asunafo North and Tano North and South), were purposively selected, in consultation with the Regional Crop Officers (Ministry of Food and Agriculture). The selection criteria were based on the production capacity and involvement of the districts in the cashew business. The sample size was estimated as 830 respondents using GeoPoll (an online sample size calculator) with the population size of 2,422,944 of the study areas, confidence interval of 3.4, and at 95% confidence level. Five major communities with good record of cashew production and its related activities in each of the nine districts were purposively sampled for the data collection.

### 2.2. Development of Questionnaire and Data Collection

Questionnaire for collecting data was developed based on three sections: demographics, knowledge and perception, and utilization of cashew apples. Both closed and open-ended questions were used to gather information from the perspectives of the respondents.

Questionnaires were administered to respondents one-on-one, and their responses to questions were then written. Five research assistants who can translate and interpret the questionnaire items in the *Twi* language (local dialect spoken by the study population) were engaged in the data collection. The questionnaire was pretested among a small group (30 respondents) of the population to test the effectiveness of it for collecting the data. After the pretesting and fine-tuning, the modified questionnaire was administered to the larger population targeting 830 respondents from top 5 cashew-growing and trading communities in each of the nine districts from the three regions. Nineteen respondents were purposively selected from each community ensuring inclusion of all the stakeholders (farmers, nut buyers, processors, and extension officers).

### 2.3. Data Entry and Analysis

Multiple questions were treated as single variables, with 1 and 2 representing yes or no as indicative of whether or not the respondents chose or had an idea of the parameter under discussion. Responses for open-ended questions were sorted, scrutinized, and categorized into thematic areas. Each theme was treated as single variable and was coded. All responses were entered into SPSS software version 20.0 and summarized into frequencies, percentages, and bar charts. Microsoft Excel 2013 was used in generating the bar charts.

## 3. Results and Discussion

### 3.1. Demographic Information of the Study Population

A total of 853 respondents comprising cashew farmers, cashew nut buyers, processors, and agricultural extension officers were interviewed. The demographic characteristics of the study population in terms of sex, age, and educational and employment status, as well as role in the cashew value chain are presented in Table 1.

Farmers form the highest (89.1%) proportion of stakeholders in the cashew industry, followed by nut buyers (6.8%), extension officers (3.5%), and cashew apple processors (0.6%). The cashew industry in Africa has been reported to mainly consist of farmers (90%) due to limited processing and other value chain activities [3, 9–11]. This is therefore not surprising that there is improvement in Ghana's share of Raw Cashew Nuts (RCN) on the global market due to high productivity from farmers, but performing poorly when it comes to cashew apple processing owning to limited processors [9]. Generally, there is a deficiency of extension officers in Ghana [12], hence the resulting outcome of having few extension officers in the cashew growing regions. This to some extent will have a greater negative impact on cashew productivity in terms of yield and quality of fruits (apples) and nuts, since agricultural extension officers are responsible for training farmers on good agronomic practices and modern technologies to maximize yield and produce quality [13]. Nevertheless, the limited number of cashew apple processors also presents business opportunities for private, government, companies, and corporate bodies to venture into.

It is very interesting to note that about 79.1% of the people involved in the cashew business are doing it as a full-time employment, suggesting continual commitment and sustainability of the industry and giving assurances for upcoming businessmen and women seeking to venture into the sector. The civil or public sector involvement in the cashew value chain is low (5%) which primarily constitutes extension officers, few active civil servant workers, and some personnel of Nation Builders Corps (NABCO) and youth employment.

The production and trading activities of cashew in the three regions (Bono, Bono East, and Ahafo) of Ghana studied are dominated by the male populace (66.2%), with female population of about 33.8%. About 41.4% and 50.5% of the study population were in the age ranges of 31-50 and above 50 years of age, respectively. These are young and energetic people forming greater percentage of the working class in the country. The future of the cashew industry in the regions and Ghana at large looks bright having quite a substantial amount of its youthful age involved in the activities of cashew. Comparatively, the good amount of women involved in the cashew industry in the study regions is an added advantage in improving their standard of living, reducing poverty, and ensuring food and nutrition security and quality of cashew since women are known to be meticulous and loyal in their dealings [14]. Dimoso et al. [15] and Ackah et al. [10] identified the cashew industry as male-dominated (54.7-74.1%), with the majority being 30-60 years of age, which is in accordance with the findings of this present study.

A greater percentage (72.3%) of people in the cashew value chain in the regions had no or basic education, and only about 15.9%, 1.2%, and 10.6% had secondary, vocational, and tertiary education, respectively. This is probably so because in Ghana and some African countries, farming is seen as a job for the less-educated ones [16, 17]. These results suggest the need to intensify educational or training programs on regular basis and also encourage well-educated ones to participate in cashew activities, in order to improve the value of the cashew industry in Ghana. The current observation is consistent with an earlier study undertaken by Ackah et al. [10] in Ghana and that of Dimoso et al. [15] in Tanzania who recorded that about 66.5% and 82%, respectively, of major cashew stakeholders (farmers) have no or basic education. Oluyole et al. [11] also revealed that the Nigerian cashew industry has about 88.2% of its workforce having no or basic formal education. The lack or low educational background of cashew stakeholders raises serious concerns about the future of the cashew industry in Ghana in terms of produce yield and handling as well as product quality and safety.

### 3.2. Stakeholders' Knowledge and Perception about Cashew Apples

Knowledge and perception about something inform its utilization and hence the need to engage the cashew stakeholders in Bono, Bono East, and Ahafo regions, the major cashew-growing regions in Ghana, to determine their knowledge and perception about cashew apples. It was evident that the stakeholders involved in cashew activities have in-depth knowledge about the health benefits of the cashew apples, and this was acquired through some informal training programs and workshops.

**Figure 1:**
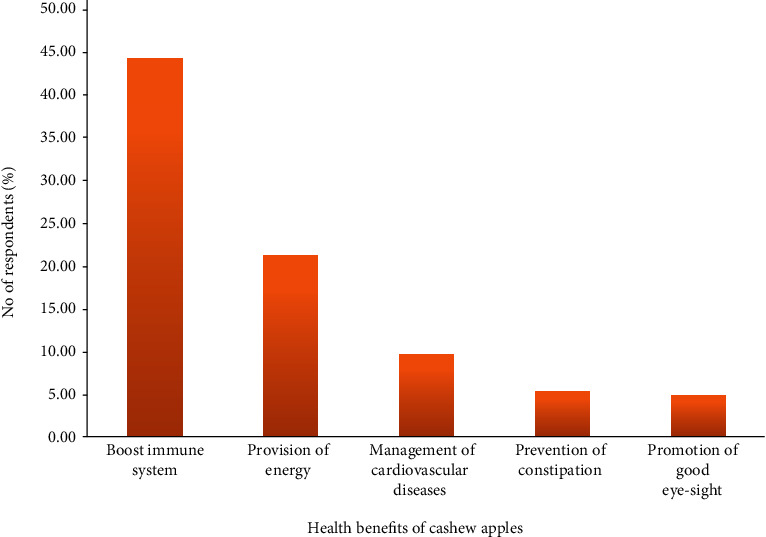
Cashew stakeholders' knowledge about health benefits of cashew apples. Note: others refer to responses outside the thematic areas.

The study revealed that about 44.2%, 21.1%, 9.61%, 5.28%, and 4.18% of the people interviewed knew the ability of the cashew apples to boost the immune system, provide energy, manage cardiovascular diseases, prevent constipation, and promote good eyesight, respectively (Figure 1). Interestingly, about 14% of the respondents also suggested other uses such as boosting sexual vitality, muscle building, wound healing, and cancer prevention among others, which correspond with the findings of Igbinadolor et al. [7] and Akinwale [6] about the health benefits of cashew apples. Some of the respondents mentioned specific nutrients like vitamin C, potassium, magnesium, and iron, which have been reported in literature to confer these health benefits [18, 19]. A study by Dimoso et al. [15] conducted in Tanzania made a contrary observation where the people had little or no knowledge on the health benefits of cashew apples. This disparity may be due to the tremendous efforts of health officials of Ghana Health Service in educating the people in the communities and training programs organized by MoFA and other NGOs like GIZ and ComCashew as indicated by the respondents.

**Figure 2:**
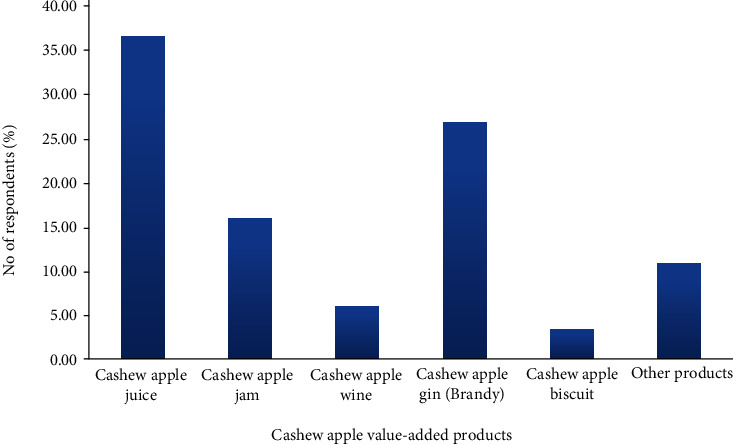
Cashew stakeholders' knowledge about the existence of cashew apple value-added products. Note: others refer to responses outside the thematic areas.

Knowledge of participants on the existence of value-added cashew apple products was tested, and the study revealed that close to about 36.46% of the respondents knew of the cashew apple juice, 26.73% of the cashew apple brandy, and 15.33% of the cashew apple jam as shown in Figure 2. Their knowledge about the existence of these products is through educational training programs and the production of cashew apple brandy by Mim Cashew and Agricultural Products Limited at Mim in the Ahafo region of Ghana. Only about 2.38% and 5.86% of the respondents were aware of the existence of cashew apple biscuit and cashew apple wine, respectively, implying that these products are new to the market; and any company going into such a business will create the niche for itself. Some products like local gin (*akpeteshie*), cashew apple syrup, and toffee were also mentioned by 10.79% of the respondents. All this information provides business opportunities for potential investors for cashew value-added products in Ghana, since the people already know or might have heard about them. A lot of value-added products from cashew apple such as jam, syrup, biscuit, juice blends, flour, wine, juice, nectar, jelly, cake, candy, fruit bar, marmalade, and animal feed have been reported [7, 9, 19, 20].

**Figure 3:**
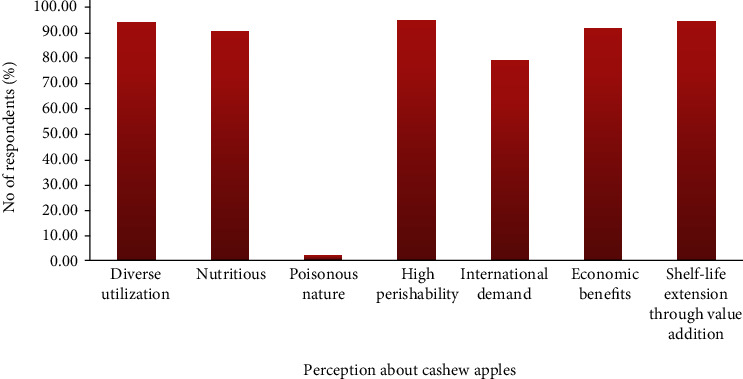
Cashew stakeholders' perception about cashew apples.

Responses to perceptions about cashew apple were very high and varied in terms of its diverse utilization (93.43%), nutritious nature (89.8%), high perishability (94.02%), shelf-life extension through value addition (93.79%), having high international demand (78.43%), and economic benefits (90.97%) with the exception of its poisonous nature recording the lowest (1.99%) as indicated in Figure 3. The lower percentage for the poisonous nature of the cashew apple is indicative of the fact that the cashew apples are safe and pose no threat to human life. The few people with the perception of cashew apple being poisonous are based on superstitious beliefs being created in the past to protect and safeguard the nuts from being taken away by passersby and also the fear of consuming with milk or sugar and microbial contamination. Some people have the perception that consumption of cashew apple with milk or sugar may result in death, which is not the case. The truth is that cashew apple has been found to contain high amount of tannins which cause milk proteins to coagulate (forming curd) when combined, and people have the notion this substance can kill [20]. It is just a perception built on no empirical data or evidence, and a study by Sousa et al. [21] has revealed that extract from cashew apple has no toxicity in zebrafish and human tumor cells. Ackah and Barreto [22] also discovered a similar outcome where greater percentage (81.1%) of the respondents said the cashew apples were not poisonous citing the same reasons.

Majority of the respondents in the present study believed that cashew apples have a lot of economic benefits and international market potential, and this can only be realized if the apples are processed into value-added products.

**Figure 4:**
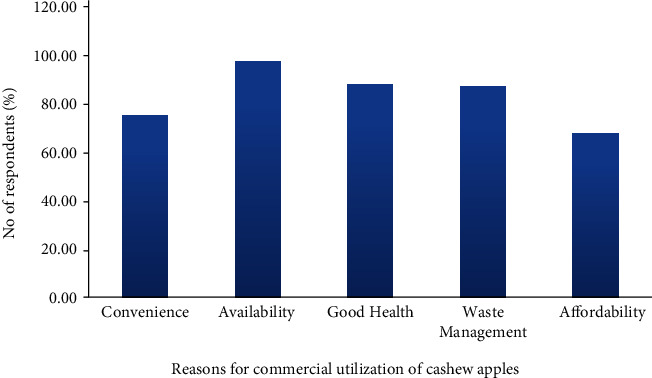
Possible reasons for commercialization of cashew apple value-added products.

The survey respondents also provided data or information on the possible reasons for the commercialization of cashew apple value-added products (Figure 4), which will serve as a basis to venture into cashew apple processing as a business. A greater percentage (97.83%) of the respondents mentioned availability as the major reason to use cashew apples or commercialize its value-added products if there are means. Although the seasonal yield of cashew apple could not be quantified, the respondents said it was in abundance when it is in season, between Late January and April. Availability of raw material is a critical issue or considerable factor during food business establishment [23], and the present study has allayed all reservations regarding cashew apple availability for commercial food production. Some of the respondents (87.46%) also had the perception that the cashew apples are nutritious and would offer a lot of health benefits if utilized. This could imply that marketing and promoting consumption of cashew apple products may not be much of a problem since the people already have some knowledge about the nutritional and health benefits of the fruit.

Also, on the issue of convenience in terms of harvesting or picking, transportation, and other handling practices, from the perspective of the respondents, about 74.44% said it is easy and convenient to undertake such activities. Some participants stated that the same or similar working/handling approaches used for other commodities like tomatoes and mangoes can be applied in dealing with cashew apples. This may indicate the willingness and commitment of stakeholders in promoting the utilization of the cashew apples or its value-added products in Ghana. However, due to its high perishability, a cold chain system would be required for large-scale processing [24].

About 86.4% of the respondents indicated waste management as a possible reason for which the apples should be put to use. Studies have shown that the cashew apple forms about 90% of the cashew fruit [3, 6], implying that a lot of waste has been generated on the farmers' fields. However, few (23.6%) of the farmers said the decaying cashew apples, to some extent, serve as manure for the land and therefore do not see that as waste.

On affordability, about 67.2% of the stakeholders in the cashew value chain mentioned that the cashew apples will be very affordable, which is quite unusual. But it also tells a story of how the cashew apples are underutilized if the majority of the stakeholders attest to its affordability and farmers' willingness to accept any amount (money) for it, although some participants (32.8%) also said the price should be moderate to augment the income of farmers. In the nutshell, there is a great market and business opportunity for interested parties to have a reasonable negotiable price for the benefit of all.

### 3.3. Utilization of Cashew Apples and Associated Challenges

**Figure 5:**
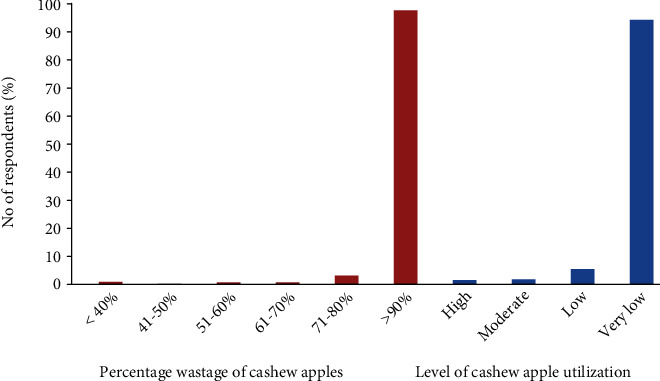
Percentage wastage and utilization of cashew apples in Ghana.

**Figure 6:**
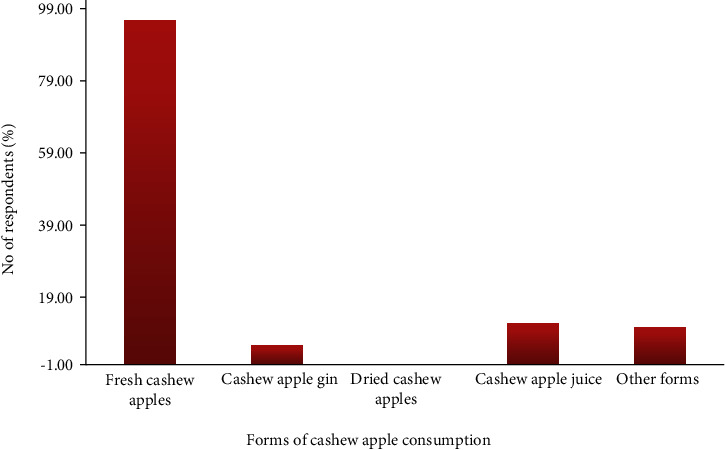
Forms of cashew apple consumption by cashew stakeholders. Note: others refer to responses outside the thematic areas.

The percentage wastage and level of cashew apple utilization are presented in Figure 5. The study revealed that more than 90% of the cashew apples go to waste as reported by approximately 95% of the respondents, which is in line with the studies by Ackah and Barreto [22], Oluyole et al. [11], and Kolliesuah et al. [9]. There is little or very low utilization for cashew apples in Ghana according to 91.7% of the stakeholders involved in the cashew business (Figure 6). Cashew apple utilization in the major cashew-growing countries has been reported to be very low (less than 10%) [3, 9, 22]. Close to 95% of the respondents disclosed that the major utilization of the cashew apples is for fresh consumption usually on the farm during harvesting, whereas 11.25% of them consume them in the form of juice. Few people (5.16%) also indicated the consumption of local cashew apple gin (brandy) due to its production by Mim Cashew and agricultural products limited based at Mim in the Ahafo region, and about 10% also had diverse utilization patterns in the form of production of mushroom, animal feed, weedicide, *akpeteshie*, soup, stew, and so on. Similar utilization of cashew apples in soups, stews, juice, jam, marmalade, and wine production was also discovered in the studies by Kolliesuah et al. [9], Oluyole et al. [11], and Ackah et al. [10].

**Figure 7:**
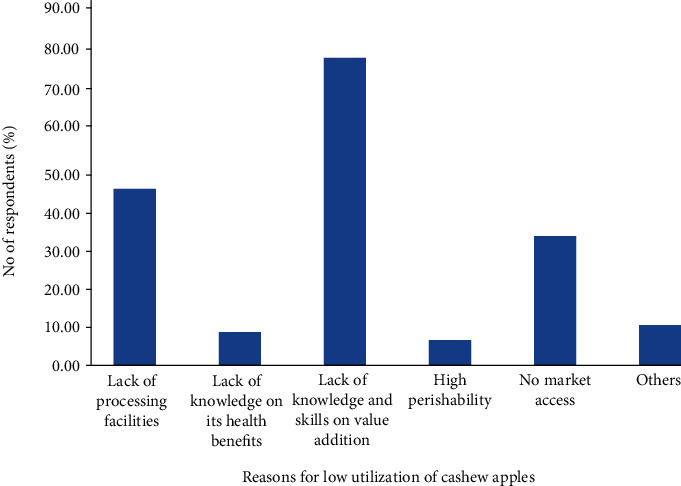
Challenges of cashew apple utilization in Ghana. Note: others refer to responses outside the thematic areas.

The high percentage wastage translates into very low utilization of the cashew apples as acknowledged by about 92% of the respondents. It was put forward by 77.02% of the respondents that this very low utilization is due to limited knowledge and hands-on skills on value addition of the cashew apples, 44.20% cited lack of processing facilities, and 32.36% indicated limited access to the market (Figure 7). These findings commensurate the results by Ackah et al. [10], Kolliesuah et al. [9], Dimoso et al. [15], and Oluyole et al. [11] which also identified lack of processing facility, knowledge or skills, and market as major constraints when it comes to cashew apple utilization. The limited knowledge or skills in the use of cashew apples makes the local people turn to rely on the government for the establishment of factories and market avenues. This presents an opportunity for training institutions and private educationists to create business or training programs to provide the necessary skills needed for value addition or processing of the cashew apples into semiprocessed or finished products.

High perishability and lack of knowledge on health benefits positioned as the key limiting factors for the utilization of cashew apples in Nigeria and Tanzania [7, 11, 15] were seen by 14.65% of the study population as minor issues inferring to their potential in handling agricultural commodities of similar or even worse physiology and deterioration and also intensified education received through the Ghana Health Service, MoFA, and other training institutions or NGOs.

**Figure 8:**
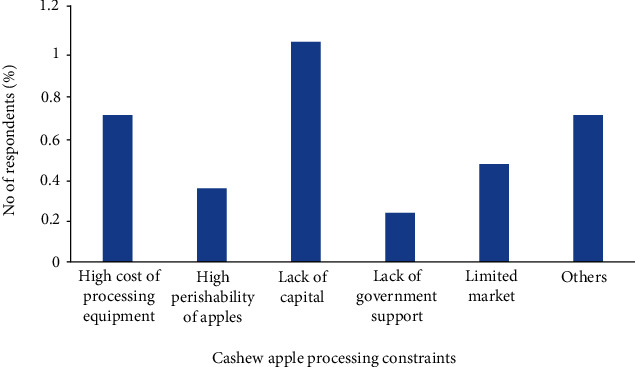
Challenges of cashew apple processors in Ghana. Note: others refer to responses outside the thematic areas.

The few processors (0.6%) in the value chain also identified high cost of processing equipment (0.7%), lack of capital (1.06%) and government commitment (0.23%), limited market (0.47%), and high perishability of the apples (0.35%) as the major issues impeding the optimum utilization of the cashew apples (Figure 8). Cashew apple processing has been characterized by challenges such as seasonal availability, high perishability, and astringency of the apples, as well as high investment cost [25, 26]. On the issue of seasonal availability and perishability, the African Cashew Alliance [27] report has indicated that storage at -17°C has been found to preserve the apples for all-year-round processing and is being practiced in Brazil. Also, studies have shown that the addition of gelatin or treatment of apple with hot water can reduce the tannin content to an appreciable level acceptable by consumers [28]. This suggests that government and NGO support of the local processors in terms of purchasing of equipment, provision of the right regulatory framework, and soft loan or access to finance for operations could maximize the level of cashew utilization in Ghana. Private sector investment and public-private partnership may also contribute significantly to the development of the cashew sector and utilization of the apples.

## 4. Conclusion

Cashew stakeholders in the major cashew-growing regions (Bono, Bono East, and Ahafo) in Ghana, consisting of farmers (89.1%), nut buyers (6.8%), extension officers (3.5%), and processors (0.6%), have in-depth knowledge about the health benefits and the numerous value-added products made from the apples. However, lack of knowledge and skills in value addition, lack of processing equipment, and limited market access are the major constraints accounting for the very low (less than 10%) utilization of the apples. The main utilization of cashew apple was its consumption as fresh fruits or juice and minor uses as an ingredient in food preparation (soup and stew), animal feed, and production of mushroom, weedicide, ethanol, and manure in rare cases.

The few processors into the processing of cashew apples identified high cost of equipment and perishability of apples and lack of capital, market, technical know-how, and government support as some challenges facing the cashew-processing companies. Stakeholders believe that processing of apples will generate extra income for farmers and create jobs and therefore call on the government to invest in the apples.

A policy aimed at equipping the stakeholders in the cashew value chain with the requisite skills needed for processing cashew apples into home-made value-added products should be promulgated. The government must invest in the processing of the cashew apples and provide stimulus packages for the private processors to expand their capacities or operations. Education on cashew apple utilization and waste reduction strategies should be integrated in the community informal training programs to enhance their knowledge level.

## Figures and Tables

**Table 1:** Demographic information of stakeholders in cashew value chain in Ghana.

Parameter		Frequency	Percentage (%)
Role in the cashew value chain	Farmer	760	89.1
	Nut buyer	58	6.8
	Processor	5	0.6
	Extension officer	30	3.5
Sex	Male	565	66.2
	Female	288	33.8
Age range (years)	<18	3	0.4
	20-30	66	7.7
	31-40	146	17.1
	41-50	207	24.3
	>50	431	50.5
Educational level	None	162	19.0
	Basic	455	53.3
	Secondary	136	15.9
	Tertiary	90	10.6
	Vocational	10	1.2
Employment status	Unemployed	28	3.3
	Self-employed	675	79.1
	Private sector	10	1.2
	Civil/public sector	43	5.0
	Student	6	0.7
	Other	91	10.7

Note: unemployed refers to respondents who have not fully been engaged in either civil or private sector. Other refers to pensioners, Nation Builders Corps, and national service personnel.

## Data Availability

Data can be obtained from the corresponding author upon request.

## References

[1] International Nuts and Dried Fruits Council (2020). Global statistical review: nutfruit magazine. https://www.nutfruit.org.

[2] Wamucii S. (2021). Ghana cashew nuts market insights. https://www.selinawamucii.com/insights/market/ghana/cashew-nuts/.

[3] Heinrich M. (2012). *Case Study of the African Cashew Initiative – Focus: Ghana*.

[4] Africa Cashew Initiative (2010). A value chain analysis of the cashew sector in Ghana. *Deutsche Gesellschaft für Technische Zusammenarbeit (GTZ)*.

[5] Catarino L., Menezes Y., Sardinha R. (2015). Cashew cultivation in Guinea-Bissau – risks and challenges of the success of a cash crop. *Scientia Agricola*.

[6] Akinwale T. O. (2000). Cashew apple juice: its use in fortifying the nutritional quality of some tropical fruits. *European Food Research and Technology*.

[7] Igbinadolor R. O., Yahaya L. E., Jayeola C. O., Adeleke S. A. (2017). Addressing the post-harvest wastages and under-utilization of cashew apple in Nigeria – a review. *The International Journal of Science & Technoledge*.

[8] Oduwole O. O., Akinwale T. O., Olubamiwa O. (2001). Economic evaluation of a locally fabricated extraction machine for a cottage cashew juice factory. *Journal of Food Technology*.

[9] Kolliesuah N. P., Saysay J. L., Zinnah M. M., Freeman T. A., Chinenye D. (2020). Trend analysis of production, consumption and export of cashew crop in West Africa. *African Crop Science Journal*.

[10] Ackah N. B., Ampadu-Ameyaw R., Appiah A. H. K., Annan T., Amoo-Gyasi M. (2020). Awareness of market potentials and utilization of cashew fruit: perspectives of cashew farmers in the Brong Ahafo region of Ghana. *Journal of Scientific Research & Reports*.

[11] Oluyole K. A., Orisasona T. M., Agbebaku E. E., Williams O. A., Abdul-Karim I. F. (2016). Evaluation of the post-harvest loss of cashew apple among cashew farmers in Nigeria. *International Journal of Applied Research and Technology*.

[12] Ghana News Agency (GNA) (2013). Inadequate extension services said to be affecting Ghana’s agric output’. https://www.ghanabusinessnews.com/2013/08/16/inadequate-extension-services-said-to-be-affecting-ghanas-agric-output/.

[13] Danso-Abbeam G., Ehiakpor D. S., Aidoo R. (2018). Agricultural extension and its effects on farm productivity and income: insight from northern Ghana. *Agriculture & Food Security*.

[14] Bachmann L. (2011). Money of her own: women’s struggle for emancipation through their dealings with money. *Journal of Comparative Research in Anthropology and Sociology*.

[15] Dimoso N., Aluko A., Makule E., Kassim N. (2021). Challenges and opportunities toward sustainable consumption and value addition of cashew apples in Tanzania. *Outlook on Agriculture*.

[16] Sumberg J., Yeboah T., Flynn J., Anyidoho N. A. (2017). Young people’s perspectives on farming in Ghana: a Q study. *Food Security*.

[17] Tadele G., Gella A. A. (2012). A last resort and often not an option at all’: farming and young people in Ethiopia. *IDS Bulletin*.

[18] Pascal A. D. C., Virginie G., Diane B. F. T. (2018). Nutritional profile and chemical composition of juices of two cashew apple’s varieties of Benin. *Chemistry Journal*.

[19] Gyedu-Akoto E. (2011). Utilization of some cashew by-products. *Nutrition & Food Science*.

[20] Poornakala S. J., Indumathi V. M., Shanthi K. (2020). Value addition in cashew apple: a review. *International Journal of Chemical Studies*.

[21] Sousa J. M. S., de Abreu F. A. P., Ruiz A. L. T. G. (2021). Cashew apple (*Anacardium occidentale* L.) extract from a by-product of juice processing: assessment of its toxicity, antiproliferative and antimicrobial activities. *Journal of Food Science and Technology*.

[22] Ackah N. B., Barreto A. L. H. (2017). *Project Report: Cashew Fruit- Adding Value for Food Security*.

[23] Park J. W., Oh H. Y., Kim D. Y., Cho Y. J. (2018). Plant location selection for food production by considering the regional and seasonal supply vulnerability of raw materials. *Mathematical Problems in Engineering*.

[24] Arah I. K., Ahorbo G. K., Anku E. K., Kumah E. K., Amaglo H. (2016). Postharvest handling practices and treatment methods for tomato handlers in developing countries: a mini review. *Advances in Agriculture*.

[25] Barve K., Vaishali A., Amit A., Barve K. (2020). *Anacardium occidentale* by-product: a review on sustainable application and added-value. *Journal of Food Nutrition and Metabolism*.

[26] Arnoldus M., Clausen B., Nikabadze G., Hanslo G., Kyd K. (2021). Cashew apple processing business models. *African Cashew Alliance*.

[27] African Cashew Alliance (No date) Cashew fruit usage in Brazil. https://www.africancashewalliance.com/sites/default/files/documents/draft_apple_processing_study_brazil.pdf.

[28] Emelike N. J. T., Ebere C. O. (2015). Influence of drying techniques on the sensory properties, physicochemical and mineral composition of beetroot juice. *IOSR Journal of Environmental Science, Toxicology and Food Technology*.

